# Treatment of Scarlet Fever

**Published:** 1845-02

**Authors:** 


### PRACTICAL MEDICINE, &c.

*Treatment of Scarlet Fever.*—At a late meeting of the Westminster Medical Society, the subject of scarlet fever and scarlatinal dropsy was discussed. Dr. Clutterbuck regarded scarlet fever as the effects of a specific poison, which would go on for a certain time and then generally subside by themselves. The duty of the practitioner would be to watch the symptons, and control them if unusually violent. It generally mattered little what was done; some mild saline, and keeping the surface cool, by sponging or cold air, was usually all that was required. If inflammation of an important organ came on, it must be treated by measures proportioned to its severity. Dropsy from scarlet fever was not common, and when it did occur, would not require active treatment. It was the result of inflammation of the skin and subjacent cellular tissue, and affected also internal organs. Occasional purgatives and mild diuretics were only required in the great majority of cases. If the tongue were coated, the pulse frequent, the skin hot, the very slight antiphlogistic treatment might be employed, but bloodletting uever, unless there were nflammation of some important organ.—*Boston Med. Journal.*

